# The process of implementation of emergency care units in Brazil

**DOI:** 10.11606/S1518-8787.2017051000072

**Published:** 2017-12-04

**Authors:** Gisele O'Dwyer, Mariana Teixeira Konder, Luciano Pereira Reciputti, Mônica Guimarães Macau Lopes, Danielle Fernandes Agostinho, Gabriel Farias Alves

**Affiliations:** I Fundação Oswaldo Cruz. Escola Nacional de Saúde Pública. Departamento de Administração e Planejamento em Saúde. Rio de Janeiro, RJ, Brasil; II Universidade Estadual do Rio de Janeiro. Faculdade de Ciências Médicas. Departamento de Clínica Médica. Rio de Janeiro, RJ, Brasil; III Fundação Oswaldo Cruz. Iniciação Científica. Escola Nacional de Saúde Pública. Departamento de Administração e Planejamento em Saúde. Rio de Janeiro, RJ, Brasil; IV Ministério da Saúde. Secretaria de Atenção à Saúde. Departamento de Ações Programáticas e Estratégicas. Brasília, DF, Brasil; V Fundação de Apoio à Pesquisa do Estado do Rio de Janeiro Iniciação Científica. Escola Nacional de Saúde Pública. Departamento de Administração e Planejamento em Saúde. Rio de Janeiro, RJ, Brasil

**Keywords:** Emergency Medical Services, Manpower, Health Manager, Health Services Administration, Resources Management, Health Management, Health Policy, Serviços Médicos de Emergência, recursos humanos, Gestor de Saúde, Administração de Serviços de Saúde, Gestão de Recursos, Gestão em Saúde, Política de Saúde

## Abstract

**OBJECTIVE:**

To analyze the process of implementation of emergency care units in Brazil.

**METHODS:**

We have carried out a documentary analysis, with interviews with twenty-four state urgency coordinators and a panel of experts. We have analyzed issues related to policy background and trajectory, players involved in the implementation, expansion process, advances, limits, and implementation difficulties, and state coordination capacity. We have used the theoretical framework of the analysis of the strategic conduct of the Giddens theory of structuration.

**RESULTS:**

Emergency care units have been implemented after 2007, initially in the Southeast region, and 446 emergency care units were present in all Brazilian regions in 2016. Currently, 620 emergency care units are under construction, which indicates expectation of expansion. Federal funding was a strong driver for the implementation. The states have planned their emergency care units, but the existence of direct negotiation between municipalities and the Union has contributed with the significant number of emergency care units that have been built but that do not work. In relation to the urgency network, there is tension with the hospital because of the lack of beds in the country, which generates hospitalizations in the emergency care unit. The management of emergency care units is predominantly municipal, and most of the emergency care units are located outside the capitals and classified as Size III. The main challenges identified were: under-funding and difficulty in recruiting physicians.

**CONCLUSIONS:**

The emergency care unit has the merit of having technological resources and being architecturally differentiated, but it will only succeed within an urgency network. Federal induction has generated contradictory responses, since not all states consider the emergency care unit a priority. The strengthening of the state management has been identified as a challenge for the implementation of the urgency network.

## RESUMO

**OBJETIVO:**

Analisar o processo de implantação das unidades de pronto atendimento no Brasil.

**MÉTODOS:**

Realizou-se análise documental, entrevistas com 24 coordenadores estaduais de urgência e um painel de especialistas. Analisaram-se questões relativas a: antecedentes e trajetória da política; atores envolvidos na implantação; processo de expansão; avanços, limites e dificuldades de implantação; e capacidade de coordenação estadual. Utilizou-se o referencial teórico da análise da conduta estratégica da teoria da estruturação de Giddens.

**RESULTADOS:**

As unidades de pronto atendimento foram implantadas a partir de 2007, inicialmente na região Sudeste, e em 2016 existiam 446 unidades de pronto atendimento considerando todas as regiões. Atualmente, há 620 unidades de pronto atendimento em construção, indicando expectativa de expansão. O financiamento federal foi um forte indutor da implantação. Os estados planejaram suas unidades de pronto atendimento, mas a existência de negociação direta entre os municípios e a União contribuiu com o significativo número de unidades de pronto atendimento construídas que não funcionam. Em relação à rede de urgência, há tensão com o hospital pela insuficiência de leitos no país, gerando internação na unidade de pronto atendimento. A gestão das unidades de pronto atendimento é predominantemente municipal, com a maioria das unidades de pronto atendimento localizadas fora das capitais e classificadas como Porte III. Os principais desafios identificados foram: o sub-financiamento e a dificuldade de contratar médicos.

**CONCLUSÕES:**

A unidade de pronto atendimento tem o mérito de ter recursos tecnológicos e ser arquitetonicamente diferenciada, mas só será bem-sucedida dentro de uma rede de urgência. A indução federal gerou respostas contraditórias, pois nem todos os estados consideram a unidade de pronto atendimento como prioritária. O fortalecimento da gestão estadual foi identificado como desafio para a implantação da rede de urgências.

## INTRODUCTION

In the early 2000s, the Ministry of Health set up a national urgency care policy, with the implementation of new components such as emergency mobile services (SAMU) and emergency care units (UPA). The pre-hospital implementation was a good decision, as national^9^ ^,^ ^12^ ^,^ ^15^ and international experiences^2^ ^,^ ^21^ show the positive impact of this care. Chronologically, the implementation of the pre-hospital components occurred separately^15^, in three different moments^10^: until 2002 - initial regulation, 2003-2008 - emphasis on the SAMU, after 2009 - emphasis on the UPA. In 2011, the need for an urgency network was defined, with regionalization and reorganization of pre-existing services^13^.

The UPA, the main fixed component of pre-hospital urgency, are intermediate units between primary care and hospital emergencies. They are classified in three different sizes, according to the population covered, the physical area, the number of available beds, the management of persons, and the capacity to care^14^.

The ordinances edited over the years have provided for UPA that are strategically integrated into urgency care networks^6^. The necessary coexistence with the SAMU and the requirement to expand the primary health care coverage with the Family Health Strategy (FHS) are conditionalities that seek to strengthen the network vision and impel managers to invest in other components of the urgency network. This is how the intended resolution of the UPA is demarcated, differentiating them from the early emergency rooms, producers of “emergency appointments” that are little resolutive^6^.

A national research^3^ has identified that all UPA classify risk and are structurally well-equipped. The main challenges found included the professional management and the “hospitalization” of patients^3^. Rio de Janeiro, the first state to implement UPA before the federal ordinances and creator of the name, built its park of units counting on a convergence of interests that has allowed its rapid expansion^9^. This state rapidly expanded the UPA without effective organization of the urgency network^7^. Another research has evidenced the limits in the management of the health work, expressed in the types of hiring, setting, and qualification of the professionals in the UPA^11^ with implications in the quality of the care. Despite the difficulties presented in the studies^7^ ^,^ ^9^ ^,^ ^11^, the UPA represented an increase in access to medical appointments and complementary tests, especially in less complex cases^7^.

The aim of this research was to analyze the implementation process of the UPA in Brazil, identifying the rules and resources that facilitated its implementation, the obstacles and facilitators in this process, the influence of the agents of the different governmental spheres in the implantation of these services, and the proposed expansion of the UPA.

## METHODS

We carried out an analysis of the official legislation, documents, and websites of the municipalities, states, and federation, as well as interviews with key players and a panel of experts.

We interviewed state emergency care coordinators who participated in a panel of experts. These players were chosen because of their importance for the integral assistance to urgencies, the negotiation of pacts and goals, and the cooperation between federative entities. In total, 24 Brazilian state coordinators agreed to participate. The interviews were based on a semistructured guide and took place in 2013 and 2014. The panel of experts collectively addressed the same subjects of the interviews, providing the opportunity to share experiences. The panel took place in two days (16 hours) at the end of 2013 and was attended by the urgency and emergency general coordinator of the Ministry of Health and three members of the Brazilian Emergency Cooperation Network (RBCE), a key institution in the formulation and implementation of the policy of urgency, in addition to the interviewed players. The material of the panel and interviews were analyzed together, considering: policy background and trajectory, players involved in the implementation, expansion process, advances, limits, and implementation difficulties, and state coordination capacity. The documents made up the rules and resources available for the action of the players involved. The analysis plan aimed to identify the structural circumstances available for the action of managers and the strategies that differentiated the states, characterizing the implementation of the UPA in Brazil.

We used the theoretical framework of the analysis of the strategic conduct of the Giddens Theory of Structuration^5^, according to which social practices can be understood as skillful procedures, methods, or techniques performed by social agents, using structural rules and resources^5^. Structural dimensions (the power base) are either facilitative or restrictive, and agents access them to manipulate and influence social interactions. There are three types of structuration, correlating the cognitive capacities of the agents and the structural characteristics: meaning, which is enabled by communication from an interpretive plan, domination, which depends on the mobilization of allocative and authoritarian resources, and legitimacy, exercised from the sanction of standards^5^.

This project was approved by CAAE 0209.0.031.000-11.

## RESULTS AND DISCUSSION

We analyzed the ordinances regarding the formulation of the Urgency Policy. The Ministry of Health provided a report that informed the sphere responsible for the management of the UPA and we consulted the webpage of the Strategic Management Support Room of the Ministry of Health, which are the main sources of descriptive quantitative data.

### History of the Fixed Pre-hospital Care

For decades, the fixed pre-hospital care was mainly assumed by municipal managers who performed this care role in different ways. Large urban centers were the precursors, with units open to spontaneous demand, but with predominant structure and equipment for low complexity care.

We highlight the emergency care services (SPA), the immediate care units (UAI), the medical emergency services (PAM), the municipal urgency and emergency centers (CMUM), and the regional health centers (CRS). The outpatient medical care units (AMA) of the municipality of São Paulo represented one of the alternatives prior to the UPA. The AMA aimed to broaden the access of the population with acute conditions of low and medium complexity to integrate them into primary care^19^. However, we identified a predominance of units with unsatisfactory integration^19^.

Before the UPA, there was a network for fixed pre-hospital that was not regulated by the Government, which fulfilled its role without systematic evaluation. Most of the time, these units were not expressive in structural terms, did not classify risk, and produced little resolutive appointments.

Although they are not pre-hospital units, small hospitals eventually had this role, especially in small municipalities and in the countryside. Unable to fulfill their function as an inpatient unit, because of deficiencies in the structure and human resources, in some places, small hospitals only provided care of spontaneous demand, confirming their idleness^20^.

The federal resource and the rule for structural definition were inductors for the UPA. However, municipal and state agents had already defined their strategies to set up the fixed pre-hospital urgency.

### Implementation and Expansion of the UPA

The implementation of the UPA began with an ordinance in 2008. The state of Rio de Janeiro was the pioneer in this process, implementing the first UPA in the country in 2007, before federal regulation. In this state, the UPA was prioritized in the governmental agenda by a confluence of historical-structural, political-institutional, and situational factors that made possible the implantation before federal incentives^9^.

According to normative definitions for the implementation of UPA, there was a requirement for the presence of a SAMU, a reference hospital network, and primary care with 50% coverage or in development, in municipalities with at least 50,000 inhabitants.

Most UPA are concentrated in municipalities with more than one million inhabitants, with the privilege of regions with better socioeconomic conditions and better provision of services, such as the Southeast region^3^.

Table 1 shows the expansion of the APU in Brazil between 2011 and 2016. We chose this period as it characterizes the beginning of federal induction from the Growth Acceleration Program (PAC). Up to 2011, the UPA were concentrated in the Southeast region. In 2016, we have an expressive number of UPA throughout the country. Of the 485 UPA in 2011 (sum of those built and under construction), 446 are ready and in operation since 2016. By 2016 there was still an expectation of expansion – 620 UPA were under construction -, despite the 132 that were built but which did not work. São Paulo has the largest park of UPA in operation, under construction, and not working. Proportionally, the North and Midwest regions have the largest number of UPA not working, considering the total in operation. The North was conservative in the implementation and expansion of UPA, with the exception of the state of Pará.

**Table 1:** Number of existing UPA, UPA under construction, and UPA built but not working, by federative unit. Brazil, 2011 and 2016.

Federative units		2011		Existing UPA	2016	UPA built but not working
		Existing UPA	UPA under construction		UPA under construction	
North Region						
	Acre	2	1	2	5	0
	Amapá	0	3	1	3	0
	Amazonas	0	9	1	4	0
	Pará*	1	20	11	40	13
	Rondônia	0	3	2	7	2
	Roraima	0	1	0	1	0
	Tocantins*	2	4	6	7	4
	Total	5	41	23	67	19
Northeast Region						
	Alagoas	0	6	8	8	2
	Bahia	1	32	29	52	12
	Ceará	0	19	26	26	9
	Maranhão	1	7	11	17	3
	Paraíba	1	6	10	18	2
	Pernambuco	14	6	18	28	7
	Piauí*	0	3	2	12	3
	Rio Grande do Norte	3	6	6	11	2
	Sergipe	0	0	4	7	0
	Total	20	85	114	179	40
Midwest Region						
	Federal District	1	8	6	4	0
	Goiás*	1	17	14	34	12
	Mato Grosso*	0	8	4	23	2
	Mato Grosso do Sul	0	4	7	7	2
	Total	2	37	31	68	16
Southeast Region						
	Espírito Santo	1	5	4	8	0
	Minas Gerais	8	43	45	47	10
	Rio de Janeiro	43	16	70	12	2
	São Paulo	13	95	99	145	19
	Total	65	159	218	212	31
South Region						
	Paraná	9	22	34	30	6
	Santa Catarina*	2	12	10	21	6
	Rio Grande do Sul*	0	26	16	43	16
	Total	11	60	60	94	28
Total Brazil		101	381	446	620	134

Source: Department of Hospital Care and Urgency (DAHU)/Ministry of Health and Strategic Management Support Room (SAGE), 2011 and 2016. UPA: emergency care units

* States with an expressive number of UPA not working, probably because some are newly built and still have no working conditions from the lack of professionals and materials.

According to the interviewees, some factors explain this scenario of varied expansion in Brazil: divergence between political interests and technical criteria, financial or operational difficulties at the municipal level, such as hiring doctors, lack of funding resources with federal payment, difficulty in the regionalization of municipal UPA, projects of UPA that later proved to be a mistake from the lack of planning, and lack of credibility of the UPA and state support.

There was unanimity about the lack of federal resources for implementation and costing, the latter theoretically shared between state and municipality.

The PAC 1 and PAC 2 were important sources of financing for the expansion of the UPA. In PAC 2, some municipalities were selected regardless of the regional urgency plans. According to the state coordinators, the attempt to regionalize the urgency was compromised when the state was removed from the expansion process, as in the following statement: “I think it was an interference that produced some expectations… the municipality says I want my own UPA… it weakened the technical part a bit… the policy that financed PAC 2 defined the expansion” (E14).

Other states were better able to act as coordinator of municipal demands: “I received several letters from the lower house requesting an UPA for the municipality… I always gave the same answer… the criterion is technical, the need of the region” (E9).

Federal induction also produced the transformation of services previously financed by states or municipalities into UPA. “The SPA (emergency care services) are 100% funded by the state. They will receive federal resource when they become UPA” (E3).

There was a coordinator who was unfavorable to the implementation of the UPA component in the urgency network, which did not prevent its implementation. Others recognized the suitability and purpose of the UPA. “I think the UPA is fundamental for the network” (E22).

The state of São Paulo illustrates a peculiarity, since its park of UPA is located outside the capital, evidencing the independence of the municipal level.

State managers were emphatic in stating that the expansion of the number of UPA, more than the initial implementation, was a federal ambition. “I think we are in a very good number. Not their expansion, but making them work is the biggest challenge with the articulation with the municipal system in which they are inserted” (E16).

The UPA, unlike SAMU^15^, did not respond to a substantive care gap for the urgency network. Its implementation depended more on the federal financial incentive, which, because it was expensive to the municipalities, produced some frustrating capacities of responses, in the conception of the managers.

The municipal manager relied on the federal entity for the expansion of the UPA and most of the state managers felt powerless in the execution of their state urgency plans.

### The Structural Conditions for the Care

The main characteristic of the UPA is the adequacy of its structure, the comfort of the facilities, and the suitability of the equipment. For the first time, a component of the SUS was proposed with great exigency in the structural criteria. This was an expressive differential, according to the interviewees, in relation to the existing emergency care park. “It evolved from the model of ER that had no resolutiveness, physical space, qualified team, material, or medication” (E7).

There was reference to the sufficiency of laboratory diagnostic resources and access to imaging resources, as observed in a national research^3^. However, the maintenance of the UPA eventually became a problem. “Lack of maintenance of the equipment ends up undermining the physical structure” (E16).

Information systems were mentioned as a structural problem. “The UPA are not computerized. They make manual reports of the care type” (E13).

However, the most striking structural issue was the lack of professionals. “The bottleneck is the human resources” (E16).

This problem had been identified nationally^3^. The low qualification of the professionals was exposed by the fact that 34% of the physicians did not have any qualification to act in the UPA. In addition, the size of the number of professionals fell short of the demand^3^. Another study has identified selection and fixation problems, predominance of young and inexperienced professionals, high turnover of physicians, and UPA seen as temporary work^11^.

The problems indicated by these studies were recognized by the interviewees, who also highlighted the incipient qualification and issues with the type of employment of professionals. “Physicians come from the FHS with no experience. Difficulty of training” (E4). “You have public workers from the municipality, borrowed from the state, those hired, it is a mixture” (E7).

The federal entity was the protagonist of a proposal of a structurally differentiated unit. However, none of the three government levels was able to positively equate the management of persons.

### The UPA and other Components of the Emergency Network

Table 1 presents and analyzes how UPA worked together with the primary care and the hospital. We highlight the following functions for the UPA: to complement primary care, to complement the hospital emergency, and to function as an inpatient unit.

The fixed pre-hospital component was regulated as an integral part of the primary care. As its implantation mainly occurred to unburden the hospitals, many managers understood the UPA as complementary to the hospital, as already evidenced^7^.

Most of the care services were classified as blue or green, that is, of low severity, as already identified in local^7^ ^,^ ^9^ ^,^ ^16^ and national studies^3^. There is no contradiction in the predominance of less severe urgencies in pre-hospital units. However, as we have already indicated^7^ ^,^ ^16^, the interviewees stated that the expectation of professionals is to treat primarily critical patients. The preponderance of less critical patients was understood as a deficiency of the primary care.

Considering that the FHS coverage in the country reached 61% in 2015, the interviewees evaluated that the UPA were not effectively related to primary care.

Some of them argued that the UPA would have less purpose if the primary care were better structured. “Dispensable if there is good primary care coverage” (E9).

Despite the impact of insufficient primary care in the UPA, the impact of the lack of hospitals is more serious, which has generated some distortions. “Some managers think that the UPA solves the shortage of hospitals” (E10).

Two serious problems can be identified in the Brazilian hospital park: a concentration of beds in small hospitals and a tendency to reduce the already undersized number of beds. The number of beds in the SUS decreased (10.5%) from 2005 to 2014^4^. In 2014, the SUS had 1.56 beds per 1,000 inhabitants, which is less than the parameter suggested by the Ministry of Health (MH) of 2.5 to three beds per 1,000 inhabitants, and which is still quite conservative^4^. “Instead of opening doors, policy should qualify them and increase beds” (E16).

Despite the mandatory hospital referral, the difficulty of hospitalizing patients was considered the greatest challenge for the UPA.

The permanence of patients for more than 24 hours in the UPA because of the lack of beds impacts the quality of care^3^. A transfer that is longer than four hours for a hospital bed aggravates the condition of the patient^8^. This long stay is a relevant issue, which reflects the difficulties of the hospital network and we highlighted it in 1, when indicating the risk of hospitalization of patients in the UPA.

In addition to the absolute deficit of beds, most of these beds are in small hospitals, with low resolution capacity and low occupancy rates. However, these beds are accounted for, despite the technical insufficiency to use them^4^. The problem is not restricted to smaller hospitals. It also reaches hospitals with more than one hundred beds, which are the main referrals for UPA. The managers knew this reality well. “We have problems with length of stay in large hospitals. We need to train the head on duty, clinical coordinators, break feuds” (E11).

The low supply of reference beds for the UPA has generated distortions. Some managers treated the more complex beds in the red room as hospital beds.

Strategies to address the problem were identified, which ranged from differentiated financial transfer up to the strengthening of the management of small and philanthropic hospitals. “There were contracts with philanthropic network beds. Resources for new beds and qualified beds” (E11).

At the federal level, initiatives to address the hospital problem are still frustrated. The consequence is overcrowding in hospital emergencies of the major hospitals of reference. This overcrowding generates pressure from the entrance^1^ and UPA may be essential because it is another open access option. Another component of overcrowding^1^, the capacity of a hospital to absorb the patient from the emergency, releasing beds, impacts the UPA, which needs this bed.[Table t2]

**Box 1 t2:** Analysis of the integration of the UPA with other components of the network.

Situation identified in the UPA	Evaluation of interviewees	Our analysis
To meet low and medium severity urgencies complementarily to primary care	The quantitative and qualitative insufficiency of the primary care produces this pattern of care.	The UPA has its function in the low severity care as it works 24 hours, complementing primary care. However, it should not be implanted to replace primary care.
"UPA meet outpatient clinic situations in most cases" (E5)		
"Patients of the UPA should be in PC. Government should decide to invest in PC" (E22)	They indicate that the noninvestment in primary care has been replaced by UPA.	
To be complementary to hospital emergency	The UPA were implemented to replace hospital emergency rooms and, in smaller municipalities, to replace inefficient hospitals.	The meeting of the low-risk demand in pre-hospital units may benefit the hospital emergency that should prioritize the more complex cases. However, that is not the purpose of the UPA.
"UPA are close to hospitals to replace the ER of the hospital" (E8)		
To work as an inpatient unit	The interviewees are against the "hospitalization" in the UPA. However, they admit that some managers count the beds of the red room as hospital beds, including ICU.	The only role of the UPA in the care of the critical patient is his or her stabilization. The UPA is 24h because it is open uninterruptedly and not because it can tolerate the patient's stay for 24 hours awaiting beds.
"UPA should not work as a hospital of low resoluteness because of the lack of beds"(E9)		
"Patient admitted to the UPA and UPA do not receive AIH" (E13)		
"UPA do well: they meet the low complexity, refer to the average, stabilize the serious condition, but hospitalization is bad" (E14)		

UPA: emergency care unit; PC: primary care; ER: emergency room; AIH: Hospitalization Authorization; ICU: Intensive Care Center

The structural difficulties of primary and hospital care did not favor the configuration of an urgency network to which the UPA was integrated.

### The Management of the UPA and the Interaction between Federated Entities

Box 2 illustrates how municipal and state players mobilized resources, legitimized, and signified the UPA as components of the urgency network, building new contexts^5^, in which the UPA can be implemented from state plans in agreement with the municipality or from autonomous municipal decision. We could see that the significance of the UPA as a structuring modality did not correspond to the expectation of a component that was integrated into the urgency network, but it meant a new access to the system. It was legitimized when it was identified as a fundamental component capable of fulfilling one of its functions, which is to be complementary to primary care. The domination structure showed that the mobilization of material and authoritative capacities generated a pattern of implementation that was sometimes more political than technical, with little state governance.

Regarding the management entity of the UPA, most of the UPA in the Brazilian regions are under municipal management, except in the Northeast. Therefore, the main institutional player of the management was the Municipal Health Department, as already evidenced^3^. The explanation has been agreements made directly between municipalities and the Ministry of Health. “We have UPA that come from the network and UPA that the mayor goes to the MH and says: ‘*I want an UPA’ and the UPA comes*" (E15)

Even the planned UPA are often not under state management by manager choice. “The state wants to assume what is from the state, the hospitals." (E15)

**Box 2:** Analysis of the structuring types and action of federative entities according to the scenario found and the statements of managers.

Category of the structuring theory		Scenario found	Statements of managers
Structuring types	Meaning - reflects an interpretive plan	UPA as a component that enables access versus UPA as a component isolated on the network	"It must give access, meet the low complexity, refer to the average, stabilize the serious condition" (E10 and E14)
			"A backset because it is isolated. It breaks the PC model" (E9)
	Legitimacy - compliance with standards	UPA as an integrated component in the network, according to ordinances	"I think the UPA is fundamental for the network" (E22)
			"Professionals understand the importance of meeting the urgency of low complexity, complementarily to PC" (E15)
	Domination - mobilization of allocative (material capacities) and authoritative resources (relation between persons)	Implementation of required units versus	"There is political articulation. The state government is requesting the UPA together with the municipality" (E11)
		Political implementation	"Policy goal of the Office of the President's Chief of Staff for 2013. It asks the MH to execute it without the state" (E12)
Agency - knowledge and action of institutional players	Action of the municipal player	Municipal managers using allocative and authoritative resources	"The municipal manager has nowhere to wash clothes, make food, and decides to put all this in the UPA, handling the financing. The manager is changed and the new one does not understand why the UPA is so big and expensive, a burden for the structure of the municipality" (E16)
			"Doctors left with the change of manager. We have thrombolytic and the new doctors don't know how to use it" (E5)
	Action of the state player	State managers carry out the urgency plan and define their management priorities and needs for the state	"State plan to open ICU beds in each health region. Consideration of the treasury" (E9)

UPA: emergency care units; PC: Primary Care; MH: Ministry of Health; ICU: Intensive Care Unit

There was report of transfer of management from the municipality to the state. One explanation was the “bankruptcy” of the municipalities in assuming the management of the UPA, being necessary to return these units to state management.

We could see the predominance of UPA located in the countryside (Table 2). A national study^3^ indicates the predominance of UPA in populous municipalities. With the current concentration in the countryside, we can infer a change in this concentration of UPA in large municipalities.

**Table 2:** Number of UPA by State, location, size, and sphere of management, 2016.

Federative units		Total of UPA	Location		Size of UPA			Sphere of management	
			Capital	Countryside	I	II	III	Municipal	State
North Region									
	Acre	2	2	0	1	0	1	0	2
	Amapá	1	1	0	1	0	0	0	1
	Amazonas	1	1	0	0	0	1	0	1
	Pará	11	1	10	2	5	4	11	0
	Rondônia	2	2	0	0	2	0	2	0
	Roraima	0	0	0	0	0	0	0	0
	Tocantins	6	3	3	1	5	0	6	0
	Total	23	10	13	5	12	6	19	4
Northeast Region									
	Alagoas	8	1	7	3	3	2	1	7
	Bahia	29	8	21	14	6	9	26	3
	Ceará	26	9	17	10	8	8	3	23
	Maranhão	11	4	7	1	7	3	1	10
	Paraíba	10	2	8	6	3	1	6	4
	Pernambuco	18	5	13	3	1	14	3	15
	Piauí	2	1	1	1	0	1	1	1
	Rio Grande do Norte	6	2	4	2	3	1	5	1
	Sergipe	4	0	4	4	0	0	3	1
	Total	114	32	82	44	31	39	49	65
Midwest Region									
	Federal District*	6		6	0	0	6	6	
	Goiás	14	2	12	4	5	5	14	0
	Mato Grosso	4	1	3	2	1	1	4	0
	Mato Grosso do Sul	7	4	3	2	2	3	7	0
	Total	31	13	18	8	8	15	25	6
Southeast Region									
	Espírito Santo	4	0	4	0	3	1	4	0
	Minas Gerais	45	6	39	11	9	25	45	0
	Rio de Janeiro	70	32	38	1	10	59	38	32
	São Paulo	99	2	97	39	41	19	99	0
	Total	218	40	178	51	63	104	186	32
South Region									
	Paraná	34	8	26	8	13	13	34	0
	Santa Catarina	10	2	8	6	1	3	10	0
	Rio Grande do Sul	16	1	15	7	5	4	13	3
	Total	60	11	49	21	19	20	57	3
Total Brazil		446	106	340	129	133	184	336	110

Source: Department of Hospital Care and Urgency (DAHU)/Ministry of Health and Strategic Management Support Room (SAGE), 2016. UPA: emergency care units

* Federal District: UPA in Brasília is classified as capital and state management to compute the sum.

The North and Midwest regions have more balance in UPA located in the capital and in the countryside. Most UPA are size III, but there is a national balance between sizes. The North region has more size II UPA, and the Northeast has more size I UPA (Table 2).

Regionalization is another fundamental strategy for the countryside where the municipalities do not meet the population criterion of 50,000 inhabitants. The sharing of resources has been a challenge. “Regional UPA do not exist because only one municipality receives resources despite the agreements made” (E8).

Another challenge is to reconcile this component with those before the UPA, with broad popular acceptance, regardless of effectiveness. “We have small hospitals in municipalities that should not exist. Maybe turn them into UPA. But they can't close the hospital in the municipality with 10,000 or 20,000 inhabitants” (E22).

The state manager builds his or her urgency plans and the municipal manager autonomously defines the priorities, but he or she suffers political influence in the defense of local interests^17^ ^,^ ^18^.

States had different opinions on the importance of UPA for their networks, and municipalities made their choice, but they often did not have the resources to maintain it.

Box 3 presents complementary statements on the categories analyzed.

**Box 3:** Additional statements of managers by analytical category.

Category	Illustrative statements	Analytical synthesis
Implantation and expansion of emergency care units (UPA)	"The value that the ministry of health and the state will give … this cost is still very high for the municipality" (E13)	Federal underfunding
	"The state is not interested. I think the pre-hospital service it has is enough and one more component, the UPA, will not change much" (E9)	Rejection of the state
	"I see the UPA as an evolved model of the emergency care that had no resolutiveness. From physical space, team qualification, equipment, medication, the UPA model is more appropriate" (E7)	Acceptance of the state
Structural conditions for care	"Well structured, they improved the working conditions of professionals" (E5)	Good structural conditions
	"We have UPA that are much better than the urgency of the hospital" (E24)	
	"Lack of pediatrician, serious problem" (E13)	Lack of medical professionals
The UPA and other components of the emergency network	"In the countryside, small municipalities have 100% of coverage of the FHS, but the doctors attends once, twice a week" (E6)	Difficulty in relation to primary care
	"The state wanted to have ICU beds in the UPA" (E5)	Lack of hospital beds
	"Mirror Hospital - metropolitan reference hospital with SH or philanthropic hospital that integrate each other to define pharmacy, bed rotation, etc." (E11)	Strategies to cope with difficulties in hospital management
Management of UPA and interaction between federated entities	"Regional UPA does not make sense. All municipalities have a small hospital that has to solve more than the UPA. The municipality ceases to invest in a service for it to invest in another regional hospital that does not have great resolutiveness. It would be different if it were a specialized regional hospital that would solve more than the municipality is capable" (E9)	Difficulty of agreement between municipalities
	"The municipality asked for an UPA in 2009, but gave up and returned the appeal. The state is resisting assuming it because the pre-hospital park it has is enough" (E6)	Difficulty of agreement between municipality and state

FHS: Family Health Strategy; ICU: intensive treatment unit; SH: small hospital

## FINAL CONSIDERATIONS

Currently, we face a project of attention to urgencies that seeks to be inclusive; however, its implementation occurred in a fragmented way and with fragile articulation among its components.

The federal normative and financial induction generated contradictory responses, in which some states consider UPA as a priority and others do not. The current number of UPA under construction needs to be reviewed to prevent the increase of UPA that do not work. The 620 UPA under construction should be analyzed in the management spaces provided for in the SUS, as the conducting groups, including the federal entity in the negotiation after planning and review of the expansion.

The strengthening of the state management was indicated as a challenge for the implementation of the network, in addition to underfunding and management of persons.

The access enabled by the UPA was beneficial, even when we consider that its demand reflects the incipience of the primary care and the hospital network. This component has the merit of having technological resources and being architecturally differentiated, but it requires investment in primary care and in hospitals to be successful and integrated into a powerful network.
